# Antilisterial Activity of Bacteriocins Produced by Lactic Bacteria Isolated from Dairy Products

**DOI:** 10.3390/foods9121757

**Published:** 2020-11-27

**Authors:** Simona de Niederhäusern, Stefania Camellini, Carla Sabia, Ramona Iseppi, Moreno Bondi, Patrizia Messi

**Affiliations:** Department of Life Sciences, University of Modena and Reggio Emilia, Via G. Campi 103/287, 41125 Modena, Italy; denieder.simona@unimore.it (S.d.N.); stefania.camellini@unimore.it (S.C.); carla.sabia@unimore.it (C.S.); moreno.bondi@unimore.it (M.B.); patrizia.messi@unimore.it (P.M.)

**Keywords:** Lactic Acid Bacteria, *Bifidobacterium*, dairy products, bacteriocin, *Listeria monocytogenes*

## Abstract

Sixty-nine Lactic Acid Bacteria (LAB) and bifidobacteria were isolated and identified from Italian dairy products (raw milk, cream, butter, soft cheese and yoghurt) to find new antimicrobial compounds to use as food bio-preservatives. All the isolates were preliminarily screened by the deferred antagonism method for bacteriocin production. Afterwards, to evaluate the release of bacteriocin in liquid medium, the Cell-Free Supernatant Fluid (CFSF) of the best producers was tested by agar well diffusion assay. The study allowed the selection of three bacteriocin producing strains (*Enterococcus faecium* E23, *Bifidobacterium thermophilum* B23 and *Lactobacillus bulgaricus* L21), endowed with the strongest and broadest inhibitory capability against the pathogen *Listeria monocytogenes*. The molecular characteristics and the chemical–physical properties of both producers and the respective bacteriocins were studied and compared. The results showed that *E. faecium* E23 was the best producer strain and its class IIa bacteriocins, called enterocin E23, exhibited a good spectrum of activity towards *L. monocytogenes*. Enterocin E23 was stable over a wide range of pH and at low temperatures for at least four months and, for this reason, it can be employed in refrigerated foods for the control of *L. monocytogenes*, the major concern in dairy products.

## 1. Introduction

Food contamination is the cause of many health and economic losses, often due to psychotropic pathogens (*Listeria monocytogenes, Aeromonas hydrophila, Yersinia* spp., etc.) [1]. In recent years this condition has been worsened by the growing trend towards the consumption of minimally processed ready-to-eat foods (RTE), lightly preserved and refrigerated products as a consequence of changes in the eating habits of consumers [2,3,4]. These new kinds of fresh foods present many safety and quality complications, especially those with extended shelf-life, more susceptible to microbial contamination for the absence, in many cases, of the adequate preservatives. The Centers for Disease Control and Prevention (CDC) has recently reported that food-borne infections cause about 76 million cases of illness, 325,000 hospitalizations and as many as 5000 deaths per year in the U.S. The economic damage caused by illness due to contaminated foods should also not to be overlooked [5]. 

The growth of spoilage microorganisms in products is another field of global concern. It is estimated that as much as 25% of all the production is lost after harvest, most of which is of poor quality due to microbial activity. These strains, like *Pseudomonas* spp., *Flavobacterium* spp., *Bacillus* spp., coliforms, etc., even if generally not harmful, have a negative impact on the shelf-life, on the organoleptic characteristics and on the overall quality of the finished products [6,7]. This usually occurs when specific spoilage organisms (SSO) grow to unacceptable levels. Their food damaging capability depends on specific storage conditions [8]. 

To inhibit and delay the growth of both pathogenic and spoilage microorganisms, chemical preservatives are frequently employed, but there are many concerns about the use of these compounds by consumers, who increasingly prefer ‘green like’ products. 

Consequently, the current trends in food processing and conservation are focusing on the use of natural preservatives, a strategy which provides for example the addition in food of microorganisms endowed with antibacterial properties or their exoproducts [2]. Lactic acid bacteria (LAB) and bifidobacteria, microorganisms generally recognized as safe (GRAS) [2,9], are good candidates for this role. They are able to extend the shelf life and ensure the safety of products [10] for their capability to minimize contamination during productive processes both by direct competition with dangerous bacteria and by production of bacteriocins. Bacteriocins are a heterogeneous group of peptides endowed with high potentiality in the food biopreservation field [11]. These antimicrobial substances are usually resistant to specific treatments (pasteurization and freezing) [12,13,14] and an increasing interest in their use in the agro-food industry is reported, in particular for lightly preserved and refrigerated foods [15,16,17].

In previous studies we reported the detection and the preliminary characterization of some bacteriocins endowed with a wide range of inhibitory spectrum produced by *Lactobacillus* and *Enterococcus* spp., isolated from meat and meat products, seafood and fresh vegetables [18,19,20,21]. The aim of the present investigation was to isolate the best LAB competitor against *L. monocytogenes*, a frequent contaminant of many foods, [22], recognized as an important foodborne pathogen and inducing agent of a serious foodborne illness, listeriosis. Dairy products are common vehicles of listeriosis responsible for human outbreaks [23] and, for these reasons, 69 bacterial strains isolated from Italian dairy products were tested for bacteriocin production. The isolation of bacteriocinogenic strains for a future use in dairy products could be advantageous in terms of biopreservation because they are well adapted to growth in this organic matrix and should therefore be more competitive than lactic bacteria from other sources. 

## 2. Materials and Methods 

### 2.1. Bacterial Strains

As part of a research program developed in collaboration with the Microbiology Laboratory of the Newlat Food S.p.A. (Reggio Emilia, Italy) a total of 69 lactic acid bacteria (LAB) and bifidobacteria were isolated and identified from Italian dairy products (raw milk, cream, butter, soft cheese and yoghurt). LAB were isolated by streaking serially diluted samples in De Man, Rogosa and Sharpe agar (MRS, Oxoid, Milan, Italy), *Lactobacillus bulgaricus* species in MRS agar and 10% acetic acid [24], *Bifidobacterium* spp. in MRS agar with the addition of L-cysteine HCl at 5%, lithium chloride at 30% and sodium propionate at 30% [25]. Cultures were incubated under anaerobic condition at 37 °C for 48–72 h. Single colonies with distinct morphological differences (color, shape and size) were randomly selected and purified on the same medium. Gram staining, catalase test and microscopic observations were performed for preliminary screening. The isolates were identified based on their biochemical properties (API 50CHL, bioMérieux France) and the five best bacteriocin producers were confirmed by by matrix-assisted laser desorption ionization (MALDI) time-of-flight mass spectrometry (TOF/MS) [26].

### 2.2. Antibacterial Activity Evaluation

Sixty-nine bacterial strains isolated from dairy products, cultured as usual in Tryptic Soy Broth (TSB) at 37 °C for 24 h, were preliminarily screened for bacteriocin production by the deferred antagonism method [27] using the same isolates as indicators. *Listeria monocytogenes* 4C, 25C and 30C from the laboratory University Collection were isolated from artisan soft cheeses and *L. monocytogenes* NCTC 10888 from National Collection Type Cultures were also employed as indicators. To eliminate inhibition due to hydrogen peroxide production the incubation was performed anaerobically. Afterwards, to evaluate the release of bacteriocin in liquid culture medium, the Cell-Free Supernatant Fluid (CFSF) of the five best producers (*Enterococcus faecium* E21, *E. faecium* E23, *E. durans* E24, *Bifidobacterium thermophilum* B23 and *Lactobacillus bulgaricus* L21) was collected by centrifugation (10,000 g for 10 min at 4 °C) and filter sterilized (0.45 mm-pore-size filter; Millipore Corp., Bedford, MA). The CFSF was tested by agar well diffusion assay [28] against the indicators found to be particularly susceptible in the initial screening, including *L. monocytogenes*.

### 2.3. Kinetic of Growth, Bacteriocin Biosynthesis and Molecular Size

To evaluate growth kinetics and bacteriocin biosynthesis, flasks (250 mL) of De Man, Rogosa and Sharpe broth (MRS Broth, Oxoid, Milan, Italy) were individually inoculated with 100 µl of *E. faecium* E23, *B. thermophilum* B23 and *L. bulgaricus* L21 at an initial cell density of about 10^5^ cfu/mL and incubated at 37 °C for 72 h without agitation. At appropriate intervals, samples were collected to evaluate the optical density and bacteriocin production. The optical density was measured by spectrophotometer using a SunRise Microplate Reader (SunRise, Tecan, Salzburg, Austria) at 630 nm (OD_630_). The antimicrobial activity was determined by agar well diffusion assay with serial twofold dilutions of the CFSF towards *L. monocytogenes* NCTC 10888. The antimicrobial titer was defined as the reciprocal of the highest dilution producing a distinct inhibition of the indicator lawn and expressed in terms of Arbitrary Units per milliliter (AU/mL) [29]. 

Lastly, to determine the molecular size of the bacteriocins, CFSFs of the producers were collected by centrifugation (12,000 g for 30 min), dialyzed against 30 mmol/l sodium acetate buffer (pH 5.3), filter sterilized (0.45 µl-pore-size filter; Millipore Corp, Bedford, MA, USA) and ultrafiltered sequentially through 30 kDa, 5 kDa mol with exclusion membranes (Diaflo Ultrafiltration Membranes, Amicron, Miami, FL, USA). Inhibitory activity was determined for both retentate and ultrafiltrate against *L. monocytogenes* NCTC 10888.

### 2.4. Sensitivity to Chemical–Physical Parameters 

In order to find the most suitable antimicrobial compound to be used as a natural preservative, the chemical–physical properties of bacteriocins produced by *E. faecium* E23, *B. thermophilum* B23 and *L. bulgaricus* L21, the only ones secreted also in a liquid medium, were compared. 

To determine the effect of temperature on bacteriocin production, cultures in TSB were incubated at 18, 37 and 40 °C for 48 h, and the antimicrobial activity was assayed towards *L. monocytogenes* NCTC 10888 by agar well diffusion.

To evaluate the sensitivity to heat and the stability during refrigerated storage, CFSFs were heated at 60, 70, 80 and 90 °C for increasing periods of time (15, 30 and 60 min), autoclaved (121 °C, 15 min) and also stored at 4 °C, and for up to four months were collected at different intervals of time and the residual antibacterial activity determined. 

The sensitivity to the proteolytic enzymes was evaluated by treating CFSFs with 0.1 mg/mL protease K (20 mg/mL) or 0.1 mg/mL pepsin or trypsin, respectively (all from Sigma Chemical Co, Saint Louis, MO, USA).

The stability to pH values was determined to adjust the CFSFs to different pH values between 3.0 and 9.0 with 1 mol/l HCl or 1 mol/l NaOH. After incubation at 37 °C for 4 h, the pH was readjusted to 6.0 and the residual antimicrobial activity determined.

### 2.5. Plasmid Curing and Transferability

To evaluate whether the bacteriocin is encoded by plasmid or chromosomal genes, the producer strains were submitted to plasmid curing treatment. *E. faecium* E23, *B. thermophilum* B23 and *L. bulgaricus* L21 were individually inoculated in TSB with increasing concentrations (1–128 µg/mL) of curing agent (ethidium bromide; Sigma Chemical Co, St Louis, MO, USA) and incubated at 37 °C for 18 h. Cultures that grew at the highest concentration of ethidium bromide were serially diluted and plated onto Tryptic Soy Agar (TSA) to obtain isolated colonies (50–80 per plate). After 24 h incubation at 37 °C the colonies were replica-plated on TSA, incubated for an additional 24 h, and examined by the deferred method for bacteriocin production. Bacteriocin producing and non-producing colonies were visually differentiated according to the presence or absence of a distinct inhibition of the indicator lawn. Immunity of non-producer variants to the antimicrobial substances was examined by agar well diffusion assay. Subsequently, plasmid DNA of *E. faecium* E23, *B. thermophilum* B23 and *L. bulgaricus* L21 and Bac- derivatives were isolated by the alkaline lysis method [30]. Electrophoresis was conducted on 0.7% agarose horizontal slab gels (10 by 7 cm, 20 cm of electrode wick distance) in Tris acetate buffer at pH 8.0 using a steady voltage of 75 V for 120 min. The purified plasmids of *Escherichia coli* V517 (54.4, 5.6, 5.1, 3.9, 3.0, 2.7 and 2.1 kb) described by Macrina et al. [31] were used as a source of size reference plasmid for molecular weight determinations.

To evaluate whether the plasmid genes that encode for bacteriocin production were transferable by conjugation, *E. faecium* E23, the producer strain with suitable chemical-physical features, was subjected to mating by the Jacob and Hobbs [32], modified method. *E. faecium* E23, nitrofurantoin and rifampicin sensitive (Bac+, Nit ^s^, Rif ^s^), was inoculated in TSB with the recipient strain *E. faecalis* JH2-2 nitrofurantoin and rifampicin resistant (Bac-, Nit ^r,^ Rif ^r^), using an initial ratio of one donor per nine recipients. The mixed cultures were incubated for 6 h at 37 °C and added to rifampicin (100 µg/mL) and nitrofurantoin (25 µg/mL). After a further incubation (8 h at 37 °C), to promote the survival of recipient and putative transconjugant cells, dilutions were plated on TSA containing the above selective agents. Following 24 h of incubation at 37 °C the Rif ^r^ Nit ^r^ colonies were replica-plated onto TSA, incubated at 37 °C for 24 h, and examined for bacteriocin production by the deferred antagonism method. The putative transconjugants were scored by the presence of an inhibition zone around the colony of indicators. 

Small-scale plasmid isolation was performed to compare the plasmid profile of the parental strains with the putative transconjugant. 

## 3. Results

### 3.1. Antibacterial Activity Evaluation

Among the 69 bacterial strains isolated from the dairy products (43 *Lactobacillus* spp., 12 *Enterococcus* spp. and 14 *Bifidobacterium* spp.) and screened by the deferred antagonism method for bacteriocin production, 30.4% (six *Lactobacillus* spp., seven *Enterococcus* spp. and eight *Bifidobacterium* spp.) presented antibacterial activity against one or more taxonomically related microorganisms. Among these, *E. faecium* E21, *E. faecium* E23, *E. durans* E24, *B. thermophilum* B23 and *L. bulgaricus* L21 showed the broadest inhibitory spectrum and an antagonistic activity towards *L. monocytogenes* strains. For this reason, the five strains have been chosen to perform further investigation. When the CFSF of these producers was tested by agar well diffusion assay, the antimicrobial activity was confirmed for *E. faecium* E23, *B. thermophilum* B23 and *L bulgaricus* L21 (Table 1). On the contrary, *E. faecium* E21 and *E. durans* E24 showed their activity by deferred antagonism only, without the release of bacteriocin in liquid culture. We called enterocin E23, thermophilicin B23 and lacticin L21 the antimicrobial compounds produced on both solid medium or liquid culture respectively by *E. faecium* E23, *B. thermophilum* B23 and *L. bulgaricus* L21.

### 3.2. Growth Kinetics, Bacteriocin Biosynthesis and Molecular Size 

**Figure 1:**
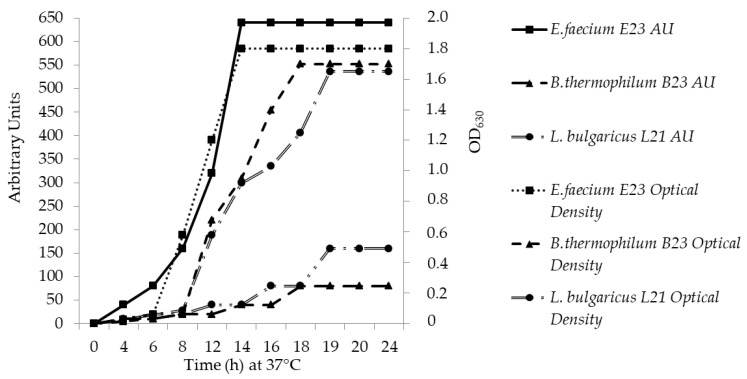
Production of enterocin E23, thermophilicin B23 and lacticin L21 at 37 °C. At appropriate time intervals, samples were taken and the bacterials growth (OD_630_) and bacteriocin activity (AU/mL) were determined.

*E. faecium* E23 and *L. bulgaricus* L21 started to produce bacteriocin after 6 h (about 40/80 AU and 20 AU), while *B. thermophilum* B23 after 8 h (about 20 AU), during the early-log growth phase, when optical density was 0.06, 0.059 and 0.063, respectively. The production of enterocin E23, thermophilicin B23 and lacticin L21 reached a maximum after 14 h (640 AU, optical density 1.8), 18 h (80 AU, optical density 1.7) and 19 h (160 AU, optical density 1.65) of incubation, respectively. Bacteriocin titer remained constant after 72 h of incubation (Figure 1).

Bacteriocin production was also affected by the different incubation temperatures. Enterocin E23 was secreted at the same concentration (640 AU) at all tested incubation temperatures (18, 37 and 40 °C for 48 h), while thermophilicin B23 and lacticin L21 showed a gradual decreasing activity up to 18° C (respectively 40 and 80 AU) (data not shown).

Lastly, with regard to the molecular size, the antibacterial activity was still found in both retentate and ultrafiltrate for enterocin E23 and only in retentate for lacticin L21 and thermophilicin B23. This suggests that the activity was linked to a compound with a molecular weight smaller than 5 kDa for enterocin E23 and greater than 30 kDa for lacticin L21 and thermophilicin B23. 

According to Klaenhammer [33], enterocin E23, a small heat-stable non-lantibiotic bacteriocin, belongs to the class IIa pediocin-like group. Antilisterial pediocin-like bacteriocins (PLB) thermophilicin B23 and lacticin L21 can instead be considered large, heat-labile antimicrobials, belonging to the class III bacteriocins.

### 3.3. Sensitivity to Chemical-Physical Parameters 

Enterocin E23, thermophilicin B23 and lacticin L21 were susceptible to the proteolytic enzymes and maintained their activity over a wide range of pH and during refrigerated storage up to four months. Concerning heat treatment, they showed a different susceptibility: whereas lacticin L21 and thermophilicin B23 appeared heat-labile and had lost the antibacterial activity against *L. monocytogenes* already after 30 min at 70 °C, enterocin E23 maintained the activity even after autoclaving (121 °C for 15 min). 

### 3.4. Plasmid Curing and Transferability

**Figure 2:**
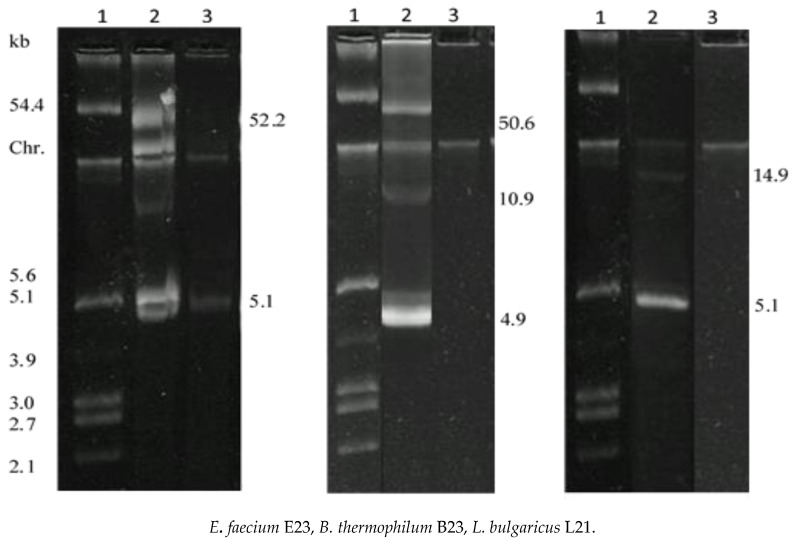
Plasmid profiles of *E. faecium* E23, *B. thermophilum* B23, *L. bulgaricus* L21 and respective Bac-derivatives after curing with ethidium bromide. Chr. denotes the position of chromosomal DNA. Lane 1: molecular size markers prepared from *Escherichia coli* (*E. coli)* V517 (54.4, 5.6, 5.1, 3.9, 3.0, 2.7 and 2.1 kb); lanes 2: wild strains Bac+, lanes 3: variant strains Bac-.

In Figure 2 the plasmid profiles of the original strains bacteriocin producers Bac+ and the respective Bac- derivatives after curing treatment were compared.

Unlike the original strain the Bac-variants showed the loss of one or more plasmids. Plasmids with different molecular weights were present in *E. faecium* E23: one large plasmid of 52.2 kb was lost in the cured Bac- variant that resulted sensitive to enterocin E23, the bacteriocin produced by the original strain. Therefore, the plasmid curing leads us to suppose that the bacteriocin phenotype could be linked to genes located on the 52.2 kb plasmid. Both *B. thermophilum* B23 and *L. bulgaricus* L21 lost all the plasmids (50.6, 10.9, 4.9 kb and 14.9, 5.1 kb, respectively) and, at the same time, the Bac- derivatives were sensitive to the bacteriocin of the original strains. Even in this case the finding suggests that the antimicrobial activity may be linked to operon located on plasmids as reported by other authors [34,35]. 

**Figure 3:**
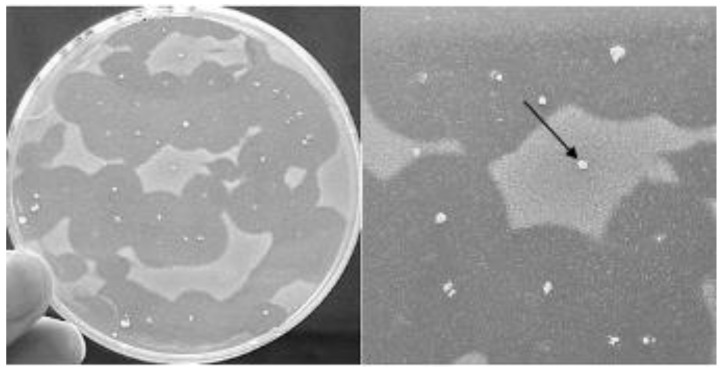
Bac- derivatives of *Bifidobacterium thermophilum* B23, indicated by arrows, were visually differentiated by deferred antagonism method according to the presence or absence of a distinct inhibition of the indicator lawn.

When the viable cells of the best producer *E. faecium* E23 were mating with the recipient plasmid free *E. faecalis* JH2-2, transconjugants bacteriocin producers *E. faecalis* JH2-E23 were generated, while no colonies were obtained in selective media with donor only, used as control (Figure 3). 

**Figure 4:**
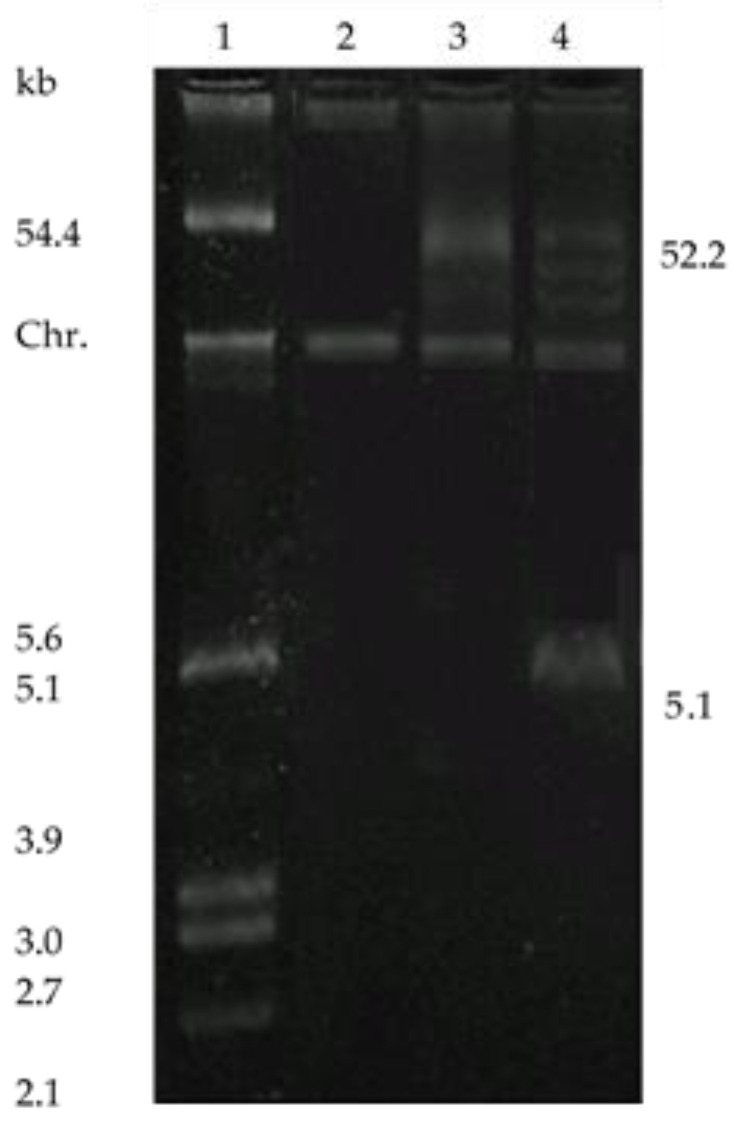
Plasmid profiles of *E. faecium* E23, *E. faecalis* JH2-2 and their transconjugants. Lane 1: molecular size markers prepared from *E. coli* V517 (54.4, 5.6, 5.1, 3.9, 3.0, 2.7 and 2.1 kb); lane 2: *E. faecalis* JH2-2 recipient strain; lanes 3: *E. faecalis* JH2-E23 transconjugant strain: lane 4: *E. faecium* E23 parental strain (52.2 kb). Chr. denotes the position of chromosomal DNA.

In Figure 4 the plasmid profile of the parental strains and their transconjugant is compared. 

The transconjugant *E. faecalis* JH2-E23, unlike the donor *E. faecium* E23, shows only the presence of the 52.2 kb plasmid, responsible for the genetic information of the bacteriocin production, as emerged from the curing treatment. This outcome confirms that this plasmid is transferable by conjugation to a bacterial specie different from the original. Lastly, it is interesting to underline that the expression of the bacteriocin in the transconjugant *E. faecalis* JH2-E23 differed from that of the parental strain, with the anti-listerial activity always overexpressed (1280-2560 AU).

## 4. Discussion

Despite the fact that hygiene and monitoring programs have been implemented in the European Union (European Communities, 2005), food-borne pathogens and spoilages bacteria, repeatedly present in raw materials, are frequently found in minimally processed ready-to-eat (RTE) foods [36,37]. The hazard associated with consumption of these products is related to the bacterial growth on food that can occur during the different stages of transformation and storage at refrigeration temperature. To prevent or limit this condition, food processing/preservation technologies are focusing on innovative strategies that include the addition to food of natural antibacterial substances. In the present work, we have studied 69 LAB and bifidobacteria isolated from Italian dairy products to obtain a compound endowed with these features. Among these, 30.4% showed antibacterial activity by deferred antagonism method against one or more taxonomically related microorganisms and, notably, *E. faecium* E21, *E. faecium* E23, *E. durans* E24, *B. thermophilum* B23 and *L. bulgaricus* L21 exhibited the best antagonistic activity towards *L. monocytogenes* strains. Only *E. faecium* E23, *B. thermophilum* B23 and *L. bulgaricus* L21 isolated from raw milk, butter and yoghurt, respectively, confirmed their activity when tested by the agar well diffusion method. This is because not all bacteriocins are secreted in liquid cultures in absence of the indicator strain that acts as promoter (activator) for its production and therefore their presence wasn’t detected in the CFSF by agar well diffusion method [38].

According to Klaenhammer classification [33], enterocin E23 belongs to the class IIa pediocin-like group, while thermophilicin B23 and lacticin L21 are belonging to the class III bacteriocins. This class is the least well characterized so far, and includes other bacteriocins of interest like helveticin M, helveticin J and enterolysin A produced by *Lactobacillus crispatus, Lactobacillus helveticus* and *E. faecalis*, respectively [39].

The plasmid analysis of the three bacteriocin producers revealed the presence of different plasmid bands, many of which were absent in the Bac- variants, obtained with the plasmid curing treatment. Since the Bac- derivatives were susceptible to bacteriocin of the original strain, this finding suggests that both antimicrobial activity and immunity phenotype are linked to genes located on the same plasmid, notably on 52.2 kb plasmid for *E. faecium* E23 as reported by other authors [34,35]. To evaluate the transferability of this plasmid, carrying the gene responsible for bacteriocin production, the producer strain *E. faecium* E23 was subjected to conjugation using the plasmid free *E. faecalis* JH2-2 as the recipient. As shown in the electrophoretic analysis, the transconjugant *E. faecalis* JH2-E23 has acquired the 52.2 kb plasmid responsible for both antimicrobial activity and immunity phenotype, as demonstrated by cure treatment. Previous mating experiments, performed using bacteriocin producer enterococci as donors and *E. faecalis* JH2-2 as the recipient, have also shown the location of genes that encode bacteriocin production in conjugative plasmids [20]. Moreover, given that the phenotypic expression of the plasmid-encoded bacteriocins can be expressed differently in hosts different from the native strains, the transferability by mating of the 52.2 kb plasmid might be considered an interesting possibility for the study of heterologous expression of bacteriocins and the development of bacterial strains with wider and improved spectrums of action. This natural mechanism of recombination can also be employed to create a multi-bacteriocinogenic strain with levels of production higher than those of the native sources [40] or to obtain a strain endowed with an amplified activity spectrum against both food-borne pathogens and spoilage bacteria [41]. A possible hurdle associated with the use of bacteriocins against these microorganisms is the development of resistant bacteria populations and this could be overcome by generating multiple bacteriocin producers [42], for example through heterologous co-production.

## 5. Conclusions

In conclusion, our study demonstrates that enterocin E23, like other bacteriocins belonging to the class IIa, exhibits an interesting antagonism against *L. monocytogenes*. The thermostability of enterocin E23 makes it suitable for use in products subjected to pasteurization treatments, to prevent the growth of both pathogenic and spoilage microorganisms, frequently isolated from food such as milk and their derivatives. This bacteriocin is also stable over a wide range of pH and may be used in acid as well as non-acid foods. Moreover, it is stable at low temperatures for at least four months and so can be employed in refrigerated foods for the control of *L. monocytogenes*, a major concern in food safety. For these features, *E. faecium* E23 and its bacteriocin enterocin E23 would be good bio-preservatives to be used in different fields of the food supply chain [43] and in particular for dairy foods. Further and more specific investigations in vivo are required to determine the efficacy and the potential benefits to the possible use of *E. faecium* E23 and its enterocin E23 in food preservation to extend its shelf-life.

## Figures and Tables

**Table 1:** Antibacterial activity of the five best bacteriocin producer strains detected in cell-free supernatant fluid by agar well diffusion assay.

		Producers *			
Indicators	E21	E23	E24	L21	B23
*Enterococcus faecalis* E11	-	++	-	++	++
*Enterococcus casseliflavus* E26	-	++	-	++	-
*Bifidobacterium* spp. B22	-	+	-	+	+
*Lactcoccus lactis* L23	-	++	-	-	++
*Lactcoccus* spp. L14	-	++	-	++	++
*Listeria monocytogene* NCTC 10888	-	+++	-	+	+
*Listeria monocitogenes* 4C	-	++	-	+	+
*Listeria monocitogenes* 25C	-	+++	-	+	-
*Listeria monocytogenes* 30C	-	++	-	+	+

Producers*: E21 Enterococcus faecium, E23 Enterococcus faecium, E24 Enterococcus durans, L21 Lactobacillus bulgaricus and B23 Bifidobacterium thermophilum. - no zone of inhibition; +, 5 mm ˂ zone ˂ 10 mm; ++, 10 mm ˂ zone ˂ 15 mm; +++, zone < 15 mm.
